# Mendelian randomization and drug repurposing analysis identifies APOE as a potential therapeutic target for low back pain

**DOI:** 10.1097/MD.0000000000050269

**Published:** 2026-08-21

**Authors:** Jianye Yang, Weihui Qi, Dong Wang, Zhenglin Mei, Hao Pan, Yongjiang He

**Affiliations:** a Department of Orthopaedics, Hangzhou TCM Hospital Affiliated to Zhejiang Chinese Medical University, Hangzhou, China; b Department of Orthopaedics, Hangzhou Ding Qiao Hospital, Hangzhou, China.

**Keywords:** low back pain, Mendelian randomization, plasma proteins, therapeutic targets

## Abstract

Low back pain (LBP) is a prevalent chronic pain syndrome primarily driven by spinal degeneration, causing persistent disability and reduced quality of life. Despite available interventions, identifying novel therapeutic targets remains imperative for modifying disease progression. We performed Mendelian randomization analyses integrating plasma proteomics data (4657 proteins in 7213 Europeans) and FinnGen R12 LBP Genome-wide association study (17,615 cases/420,066 controls). Robustness was confirmed via Bayesian colocalization and Steiger directionality tests. Protein-protein interaction networks, functional enrichment (Kyoto Encyclopedia of Genes and Genomes/gene ontology), single-cell annotation, and druggability assessments were subsequently applied. Mendelian randomization identified Apolipoprotein E (APOE) as causally protective against LBP (OR = 0.86, 95% CI: 0.81–0.91, *P* = 5.12 × 10^−7^). Bayesian colocalization supported shared causal variants, while the Steiger test excluded reverse causation. Protein-protein interaction networks revealed interactions between APOE and established LBP drug targets. Functional analyses demonstrated APOE’s enrichment in lipid metabolism pathways (particularly cholesterol transport), with elevated expression in macrophages/fibroblasts and hepatic/cerebral tissues. Druggability assessment indicated: Glucocorticoids directly upregulate endogenous APOE; Inflammatory cytokine receptor antagonists and PPARγ agonists indirectly enhance APOE activity. Genetically elevated APOE expression reduces LBP risk, establishing its therapeutic potential. Pharmacological modulation of APOE via glucocorticoids, cytokine antagonists, or PPARγ agonists represents a novel strategy for early intervention in disc degeneration.

## 1. Introduction

Low back pain (LBP) is a prevalent condition characterized clinically by pain in the lower spinal region. Based on etiological features, LBP can be classified into 2 primary categories: specific low back pain and nonspecific low back pain. Notably, nonspecific low back pain accounts for approximately 80% to 90% of clinical cases, representing the predominant form of LBP.^[1,2]^LBP is currently one of the leading causes of disability worldwide; it severely impacts patients’ quality of life while simultaneously imposing a substantial economic burden on society.^[3,4]^Current clinical management of LBP primarily employs multimodal comprehensive intervention strategies, including surgical, pharmacological, and exercise rehabilitation therapies; in recent years, complementary and alternative medicine approaches such as acupuncture have gained increasing application.^[5–8]^However, due to the significant etiological heterogeneity and diverse pathophysiological mechanisms underlying LBP, not all therapeutic approaches are effective across different patient subgroups. Thus, diversifying therapeutic strategies and identifying novel drug targets for LBP remain critically important for improving patient outcomes.

Plasma proteins, as key functional elements in blood, play vital roles in regulating biological processes. Alterations in the expression levels and activity of specific plasma proteins are closely linked to the pathogenesis and progression of various diseases. During disease advancement, these proteins often exhibit characteristic alterations in concentration and function. Such dynamic patterns of change render them promising candidates for therapeutic intervention targets.^[9]^Research indicates that during the development and progression of LBP, proteins released from damaged cells gain access to the circulatory system, leading to characteristic alterations in plasma protein composition and biological activity.^[10]^Systematic characterization of the causal links between these characteristic plasma protein alterations and LBP will enable the identification of potential therapeutic targets for LBP.

Mendelian randomization (MR) is a causal inference methodology leveraging genetic variation, which establishes causal relationships between exposures and clinical outcomes by utilizing single nucleotide polymorphisms (SNPs) as instrumental variables (IVs). In recent years, owing to its capacity to effectively overcome confounding factors inherent in observational studies, MR has evolved into a vital research tool for the discovery and validation of disease therapeutic targets, gaining widespread adoption in biomedical research.^[11]^Its core principle rests on Mendelian inheritance laws and encompasses 3 fundamental assumptions: the relevance assumption, which requires that genetic instruments must be strongly associated with the exposure; the independence assumption, which mandates that genetic instruments are independent of confounding factors; and the exclusion restriction assumption, which stipulates that genetic instruments influence the outcome exclusively through the exposure.^[12]^By leveraging MR, we can effectively circumvent interference from confounding factors prevalent in conventional studies, thereby significantly enhancing the specificity and robustness of drug target identification.^[13]^

**Figure 1.**
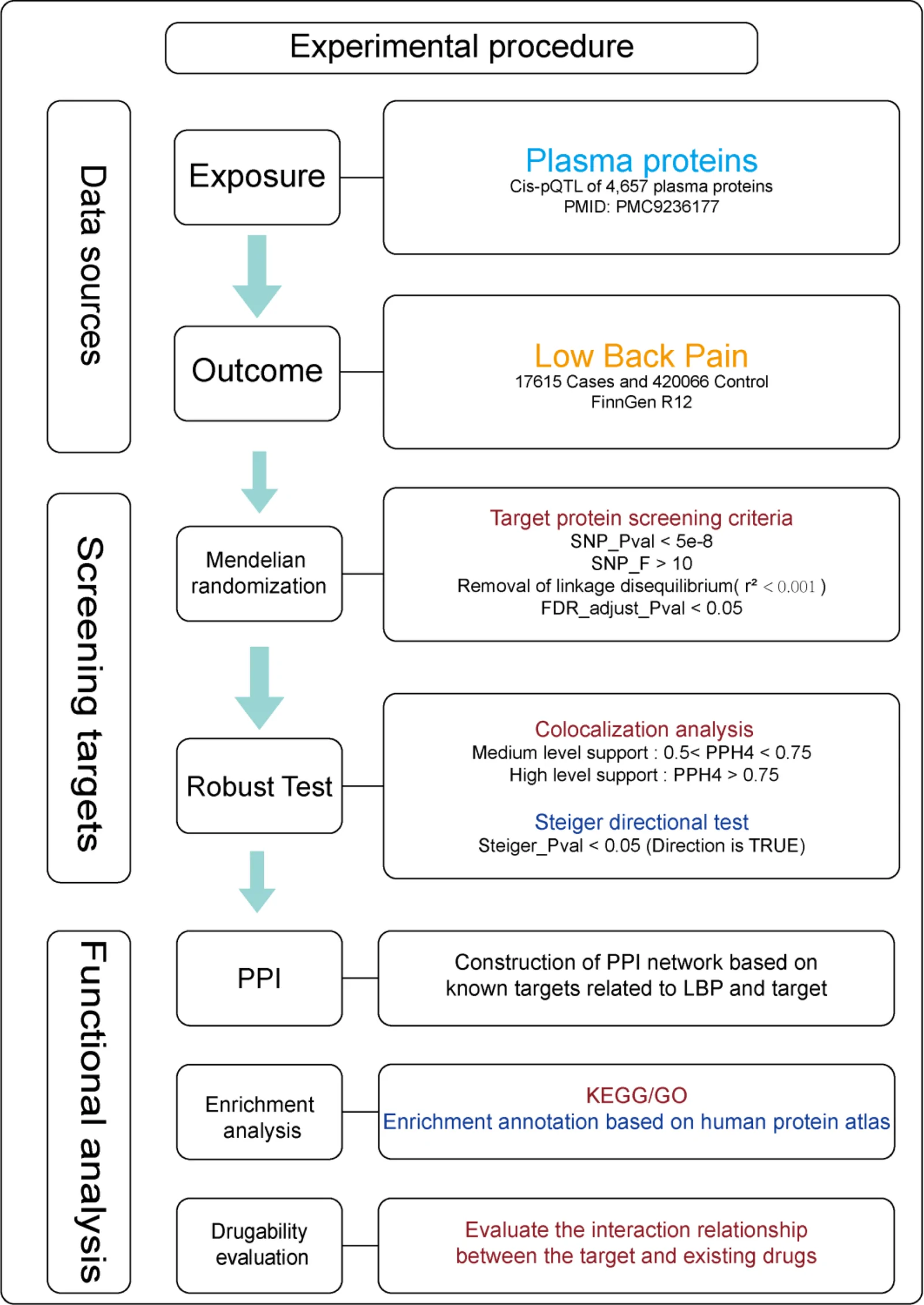
Experimental procedure. The flowchart illustrates the sequential analytical pipeline. First, cis-protein quantitative trait loci (cis-pQTLs) for 4657 plasma proteins were extracted from a large-scale European-ancestry proteomics study and used as instrumental variables (IVs). Second, two-sample Mendelian randomization (MR) analysis was performed against the FinnGen low back pain (LBP) GWAS summary statistics (17,615 cases and 420,066 controls), followed by false discovery rate (FDR) correction. Third, significant protein candidates underwent Bayesian colocalization and Steiger directionality testing to confirm robustness and causal direction. Fourth, protein-protein interaction (PPI) networks, KEGG/GO functional enrichment, and single-cell/tissue annotation were conducted. Finally, systematic druggability evaluation was performed via the DGIdb database and literature mining to identify potential pharmacological modulators. GO = gene ontology, KEGG = Kyoto Encyclopedia of Genes and Genomes.

In this study, we designed a two-sample MR analytical framework, utilizing human plasma protein quantitative trait loci (pQTLs) as the exposure and LBP as the outcome, to elucidate causal relationships between plasma proteins and LBP and simultaneously identify significantly differentially expressed proteins during LBP pathogenesis as potential therapeutic targets. To ensure result robustness, we further implemented Bayesian colocalization analysis and Steiger directionality testing. Following target screening, we constructed a protein-protein interaction (PPI) network between candidate proteins and known LBP-associated targets to delineate their molecular interactions. Concurrently, through integrated Kyoto Encyclopedia of Genes and Genomes (KEGG) pathway analysis, gene ontology (GO) functional annotation, and single-cell/tissue-specific enrichment analysis, we comprehensively characterized the biological functions and spatial distribution patterns of these target proteins across cell types and tissues, providing a critical foundation for subsequent drug development. Finally, we conducted a systematic druggability evaluation of core target proteins to holistically assess their development potential as therapeutic targets for LBP. (Fig. 1) The design, analysis, and reporting of this study followed the TITAN guidelines for transparency in artificial intelligence reporting to ensure methodological rigor and reproducibility of results.

## 2. Materials and methods

All data utilized in this study were obtained from previously published sources or public databases. Having received ethical approvals and informed consent during their original collection, no additional ethics approval was required for the current analyses.

### 2.1. Data source for plasma protein cis-pQTLs

Plasma protein cis-pQTL data were obtained from a large-scale integrated study by Zhang et al.^[14]^This study aggregated and analyzed cis-pQTL data for 4657 plasma proteins from 7213 individuals of European-ancestry. These data served as exposure variables for subsequent two-sample MR analyses. In that study, protein levels were first residualized for covariates including sex, age, genetic principal components, study sites, and PEER factors, and then normalized by quantile-quantile transformation. Accordingly, the causal estimates from our MR analysis reflect the effect of a 1 standard deviation (SD) increase in the genetically predicted level of each plasma protein on LBP risk. These data served as exposure variables for subsequent two-sample MR analyses.

### 2.2. LBP data source

Summary statistics for LBP were derived from the FinnGen database (Release R12), a large-scale Genome-wide association study (GWAS) repository comprising 500,000 Finnish individuals.^[15]^We extracted LBP outcome data using the keyword “Low back pain” in FinnGen, yielding a sample of 437,681 individuals (17,615 cases and 420,066 controls). Diagnostic codes for LBP in the primary MR analysis followed the International Classification of Diseases, 10th edition (ICD-10), including: M54.5 (Low back pain) and M51.1 (Lumbar and other intervertebral disc disorders with radiculopathy). It is important to note that this combined phenotype encompasses both nonspecific LBP and more structurally-defined disc disease with radiculopathy, representing a broad LBP outcome in our primary analysis.

### 2.3. Selection of IVs

The study applied stringent criteria to screen plasma protein cis-pQTLs as IVs, including: Genome-wide significance: SNPs significantly associated with target plasma proteins (*P* < 5 × 10^−8^); Inkage disequilibrium independence: Ensured SNP independence (*r*^2^ < 0.01, clumping window = 10,000 kb); Strong instrument validation: Excluded weak IVs (*F*-statistic>10); Data harmonization and quality control: During the harmonization of IVs between the exposure and outcome datasets, we ensured allele alignment and excluded mismatched or ambiguous SNPs. Specifically, we removed palindromic SNPs and any variants that were missing from either the exposure or outcome summary statistics.

### 2.4. Proteome-wide MR analysis

Given variations in the number of available SNPs across screened proteins, we employed 2 complementary MR methods to assess causal effects: the Wald ratio method for proteins with only 1 valid SNP as the IV; Inverse-variance weighted regression as the primary analysis for proteins with ≥2 SNPs.^[16]^To mitigate false positives from multiple testing, we applied false discovery rate (FDR) correction to preliminary MR results. Plasma proteins with FDR-adjusted *P* < .05 (Benjamini-Hochberg method) were defined as significantly causally associated with LBP.

### 2.5. Bayesian colocalization analysis

To further validate the reliability of MR findings, we performed Bayesian colocalization analysis on plasma proteins significantly associated with LBP. This method determines whether 2 traits (here, plasma protein levels and LBP) share a single causal genetic variant within a specific genomic region by calculating the posterior probability (PPH). A high PPH value indicates that the target protein and LBP likely share the same causal variant at the examined locus.^[17]^This method evaluates 5 mutually exclusive hypotheses: H0: No causal variants for either plasma proteins or LBP in the genomic region; H1: Causal variants for plasma proteins only; H2: Causal variants for LBP only; H3: Distinct causal variants for plasma proteins and LBP (coincidental colocalization); H4: A single shared causal variant influencing both traits (true colocalization). Among these, PPH4 serves as the key diagnostic metric, quantifying the probability that plasma protein levels and LBP share an identical causal genetic variant. Interpretation thresholds: PPH4 >0.75 indicates strong evidence for shared genetic causality, while 0.5 <PPH4 <0.75 suggests moderate support for shared causality.^[18]^

### 2.6. Steiger directionality test

To eliminate potential reverse causation bias, we performed Steiger directionality testing for target proteins. This method evaluates the reliability of the “plasma protein → LBP” causal direction by comparing phenotypic variance explained (R^2^) by instrumental variables (SNPs) between the exposure (Plasma proteins) and outcome (LBP). When the variance explained by IVs is significantly greater for plasma proteins (R^2^.exposure) than for LBP risk (R^2^.outcome) (*P* < .05), the causal pathway “plasma protein → LBP risk” is supported. By excluding the possibility of reverse causation (“LBP risk → Plasma protein”), this approach effectively controls for directional bias and significantly enhances the reliability of MR conclusions.^[19]^

### 2.7. Functional pathway enrichment analysis

We performed pathway enrichment analysis of target proteins using the SangerBox platform, incorporating both the Kyoto Encyclopedia of Genes and Genomes (KEGG) and GO databases. Additionally, cellular and tissue enrichment annotations for target proteins were retrieved from the Human Protein Atlas database to characterize their distribution patterns in human biological systems.

### 2.8. Protein-protein interaction network construction

Based on MR analysis and sensitivity testing results, we selected 7 key target proteins: MMP13,^[20]^ IL-6,^[21]^ IL-1β, TNF-α,^[22]^ LCN2,^[23]^ SIRT1^[24]^ and NGF^[25]^: along with the screened candidate proteins for PPI network construction. These key targets have been previously reported as LBP-related biomarkers, most of which are closely associated with intervertebral disc degeneration (IDD), the primary pathological basis of LBP. Using the STRING database with a stringent high-confidence threshold (interaction score ≥0.7), we constructed the PPI network to investigate potential functional relationships between the candidate proteins and these established biomarkers, thereby evaluating their likelihood of influencing LBP pathogenesis through regulatory networks.

### 2.9. Druggability evaluation

To assess the therapeutic potential of target proteins, we conducted a comprehensive analysis using the DGIdb database (v5.0.3) to identify approved or clinically investigated targeted drugs. This was complemented by systematic literature mining to integrate the latest research evidence. By synthesizing bioinformatics data with preclinical/clinical findings, we performed a multidimensional evaluation of each target protein’s feasibility as an LBP therapeutic target, with particular focus on drug specificity toward Apolipoprotein E (APOE) modulation and mechanistic evidence underlying pharmacological effects. This integrated approach provides an evidence-based foundation for subsequent target development.

### 2.10. Statistical analysis

All MR analyses were conducted using the R programming language (version 4.5.0). The “TwoSampleMR” package was employed to estimate causal effects between exposures and outcomes, while the “COLOC” package was utilized for Bayesian colocalization analysis.

## 3. Results

### 3.1. MR analysis results

Under stringent screening criteria, this study incorporated 2825 SNPs corresponding to 1619 plasma proteins as IVs. Empirically confirmed, all IVs exhibited *F*-statistics >10, indicating an absence of weak instrument bias and fulfillment of core MR assumptions (Supplementary Table 1, Supplemental Digital Content 1).

**Figure 2.**
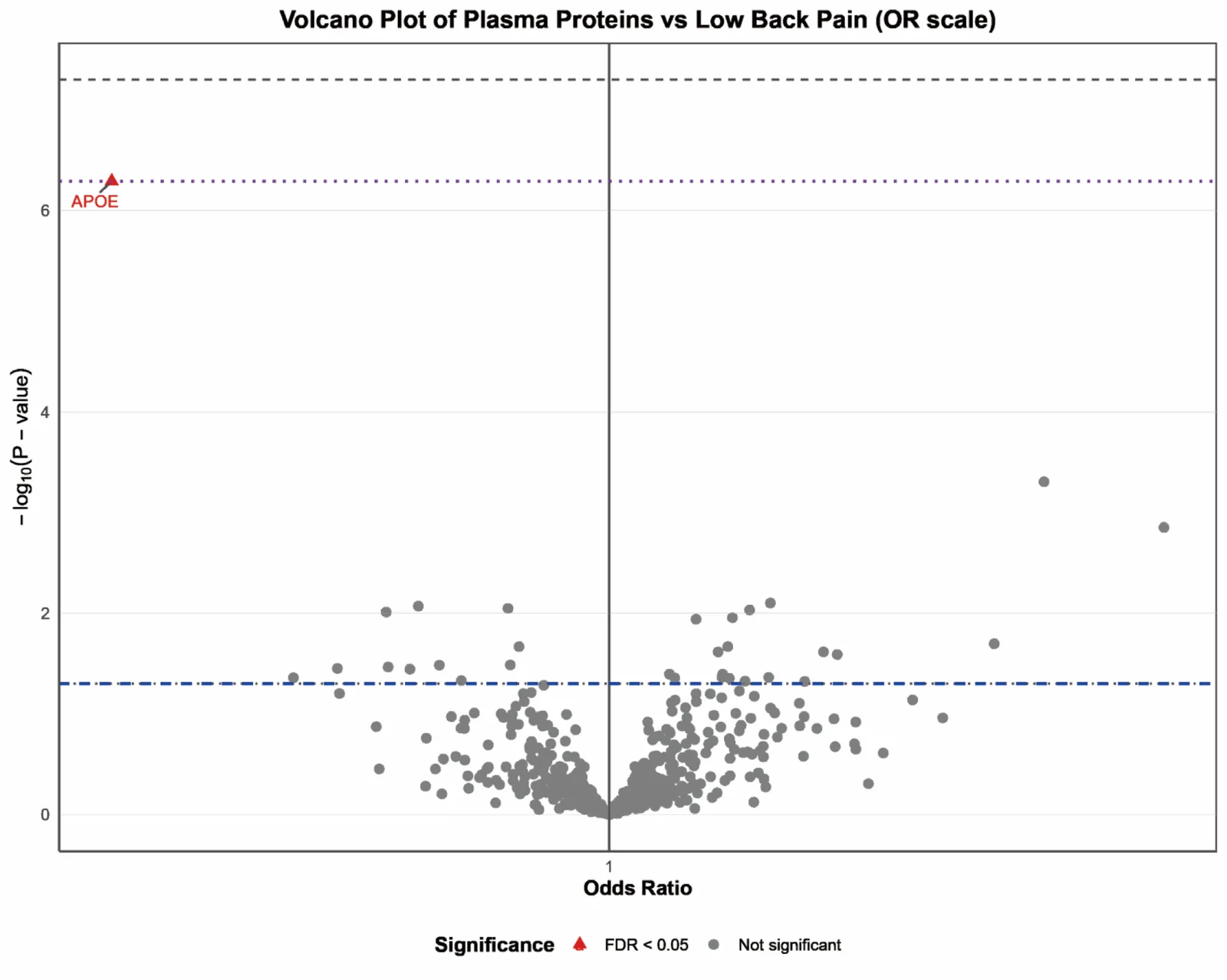
Volcano plot of proteins after FDR correction. The volcano plot displays the results of the two-sample Mendelian randomization analysis for 1619 plasma proteins. The x-axis represents the odds ratio (OR) for LBP per 1 standard deviation increase in genetically predicted protein levels (log-transformed), and the y-axis represents the statistical significance (−log10 *P*-value). The blue dashed horizontal line indicates the nominal significance threshold (*P* = .05), while the purple dashed horizontal line indicates the significance threshold after Benjamini-Hochberg FDR correction (FDR-adjusted *P* = .05). Each point represents an individual plasma protein. The red inverted triangle highlights Apolipoprotein E (APOE), which was the only protein that remained statistically significant after FDR correction (OR = 0.86, 95% CI: 0.81–0.91, *P* = 5.12 × 10^−7^), suggesting a protective causal effect.

We employed rigorously screened SNPs as IVs and utilized GWAS summary statistics for LBP cases from the FinnGen database as outcome variables to conduct two-sample MR analyses. To mitigate false positive risk from multiple testing, all MR results underwent FDR correction via the Benjamini-Hochberg method. Initial two-sample MR analysis revealed that among the 1619 plasma proteins analyzed using the inverse-variance weighted method, 32 demonstrated statistically significant associations with LBP (*P* < .05) (Supplementary Table 2, Supplemental Digital Content 2), suggesting potential causal relationships. Following FDR correction, only APOE retained statistical significance (FDR-adjusted *P* < .05) among these candidate proteins. MR analysis demonstrated a significant protective association between APOE and LBP, with the OR corresponding to a 1 SD increase in genetically predicted plasma APOE levels (OR = 0.86, 95% CI: 0.81–0.91; *P* = 5.12 × 10^−7^), indicating that elevated APOE expression may reduce LBP risk. Although the remaining 31 proteins did not survive multiple testing correction (FDR-adjusted *P* ≥ .05), they exhibited potentially suggestive associations that may provide valuable leads for future investigations (Fig. 2).

### 3.2. Bayesian colocalization analysis results

**Figure 3.**
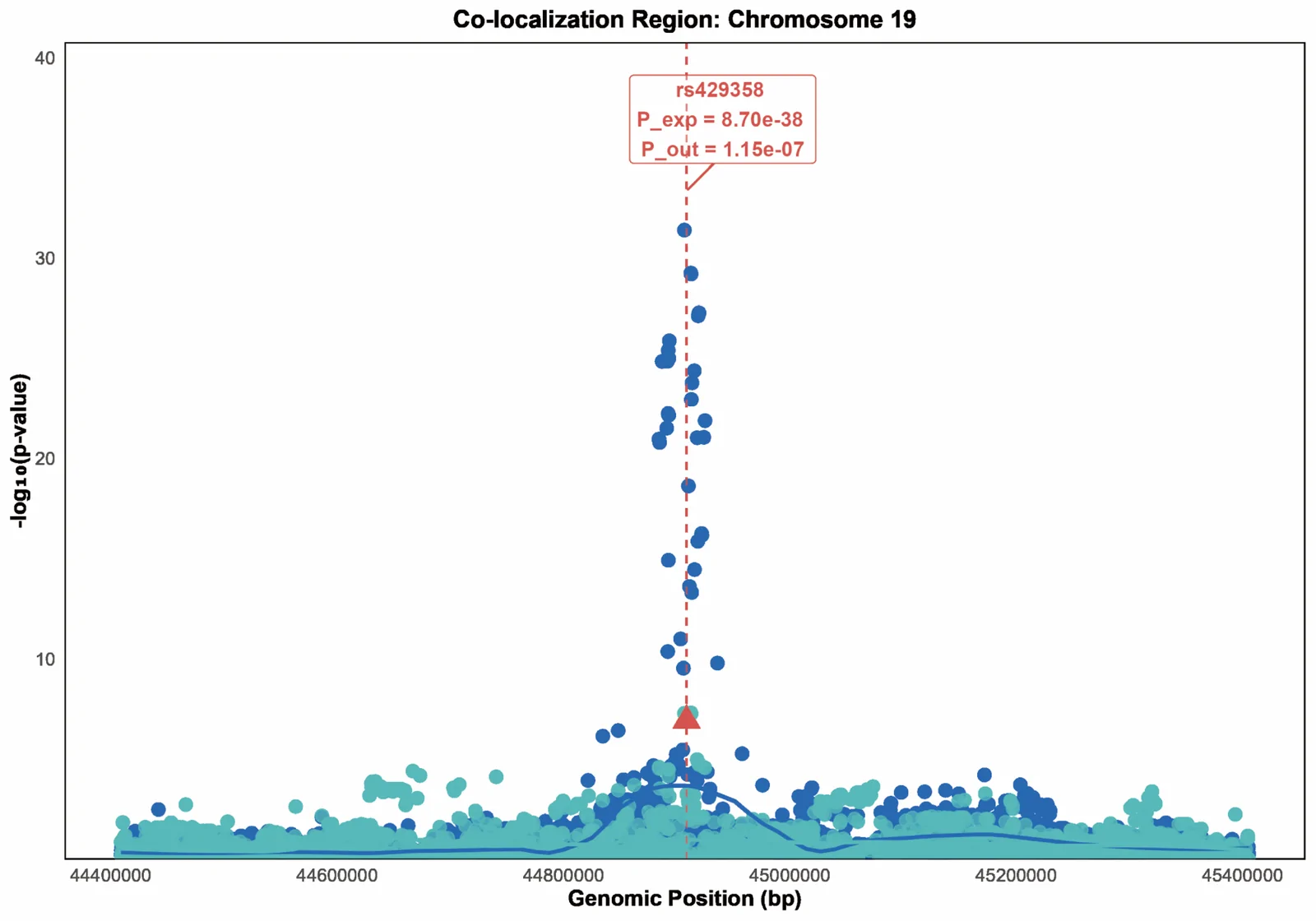
Colocalization Analysis of APOE and LBP. The plot shows the Bayesian colocalization analysis results across the genomic region of the APOE gene locus (chromosome 19). The x-axis represents the genomic position (physical map), and the y-axis represents the −log10 *P*-value for genetic associations. The upper panel displays association signals for plasma APOE protein levels (pQTL), and the lower panel displays association signals for LBP (GWAS). Each dot represents a single nucleotide polymorphism (SNP), color-coded by linkage disequilibrium (LD) with the lead sentinel variant (red diamond in the center). The high overlap of association signals between the 2 traits provides strong support for a shared causal variant (posterior probability for hypothesis 4, PPH4 > 0.75), confirming the robustness of the MR findings. CI = confidence interval, FDR = False discovery rate, OR = odds ratio.

To validate the reliability of the causal association between APOE and LBP, we performed Bayesian colocalization analysis. The results provided strong evidence supporting a shared causal genetic variant underlying both traits, based on the evaluation of 3090 variants within the APOE genomic region (Table 1), thereby confirming the robustness of the MR findings (Fig. 3).

**Table 1:** Results of Bayesian colocalization analysis for APOE and LBP.

Protein	Nsnps	PP.H0.abf	PP.H1.abf	PP.H2.abf	PP.H3.abf	PP.H4.abf
APOE	3090	1.0007 × 10^−34^	0.0004	1.9848 × 10^−33^	0.0070	0.9926

The table presents the posterior probabilities (PPH) for 5 mutually exclusive hypotheses evaluated using the COLOC method across 3090 variants in the APOE genomic region. H0: no causal variant for either trait; H1: causal variant for APOE only; H2: causal variant for LBP only; H3: distinct causal variants for both traits (coincidental colocalization); H4: a single shared causal variant for both traits (true colocalization). The high PPH4 value (>0.75) indicates strong evidence that both plasma APOE levels and LBP share the same underlying causal genetic variant, supporting the MR findings.

### 3.3. Steiger directionality test results

APOE underwent Steiger directionality testing (Table 2). The results excluded the possibility of reverse causation (LBP→APOE), further validating the reliability of APOE as a protective factor against LBP through causal inference and providing directional assurance for target discovery.

**Table 2:** Results of Steiger directionality test.

Exposure	Outcome	R^2^.exposure	R^2^.outcome	Correct_direction	Steiger_*P* val
APOE	LBP	0.0127	4.6741 × 10^-6^	TRUE	.0148

The table shows the variance in phenotype explained (R^2^) by the instrumental variables for the exposure (plasma APOE) and the outcome (LBP). The Steiger test evaluates the causal direction by comparing these R^2^ values. A significant Steiger *P*-value (<.05) indicates that the instrumental variables explain significantly more variance in the exposure (APOE) than in the outcome (LBP), thereby excluding reverse causation (LBP → APOE) and confirming the causal direction of “plasma APOE → reduced LBP risk”.

### 3.4. Functional enrichment analysis results

**Figure 4.**
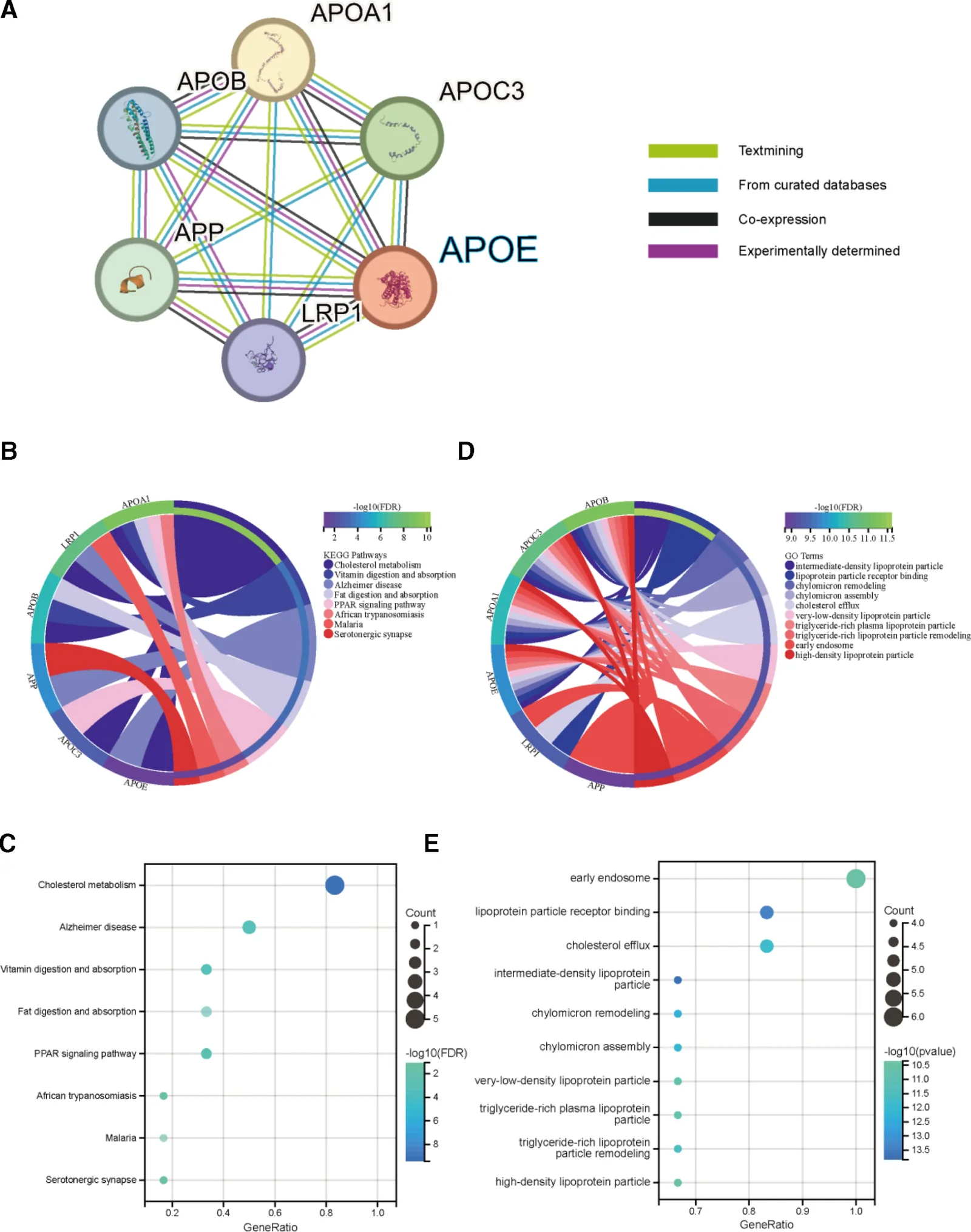
APOE-associated proteins and KEGG/GO enrichment analysis results. (A) Protein-protein interaction (PPI) network of proteins directly interacting with APOE, constructed using the STRING database with a high-confidence interaction score threshold >0.90. Nodes represent proteins, and edges denote predicted functional associations. (B) KEGG pathway enrichment circle plot showing the top significantly enriched pathways, with the outer ring linking genes to pathways and the inner ring indicating gene counts. (C) KEGG enrichment bubble plot where the x-axis represents the gene ratio and the y-axis represents the enriched pathways, with bubble size indicating gene count and color representing significance level. (D) Gene Ontology (GO) enrichment circle plot illustrating the top biological processes, cellular components, and molecular functions. (E) GO enrichment bubble plot detailing the top significant terms. The analyses collectively highlight APOE’s predominant enrichment in cholesterol metabolism and lipoprotein transport pathways. KEGG = kyoto encyclopedia of genes and genomes.

Because proteome-wide MR prioritizes putatively causal circulating proteins, integrating downstream network-based interpretation (PPI) and pathway enrichment provides a biologically grounded hypothesis framework and highlights targets that can be further triangulated with multi-omics evidence to strengthen mechanistic inference and translational prioritization.^[26,27]^Based on the significant association between APOE and LBP identified by MR analysis, we performed systematic functional annotation. Initial screening via the STRING database (high-confidence threshold >0.90) revealed a protein interaction network strongly associated with APOE. KEGG/GO enrichment analysis demonstrated significant enrichment of APOE and its interacting proteins in cholesterol metabolism pathways, particularly lipoprotein assembly and transport processes. Single-cell enrichment annotation indicated cell type-specific enrichment of APOE in macrophages and fibroblasts. Tissue distribution profiling showed predominant APOE expression in the liver, followed by brain and adrenal tissues, with enrichment also observed in skeletal muscle. These multi-omics characteristics collectively elucidate APOE’s critical biological role and provide a systematic biological foundation for its potential as an LBP therapeutic target (Fig. 4).

### 3.5. Protein-protein interaction network analysis results

**Figure 5.**
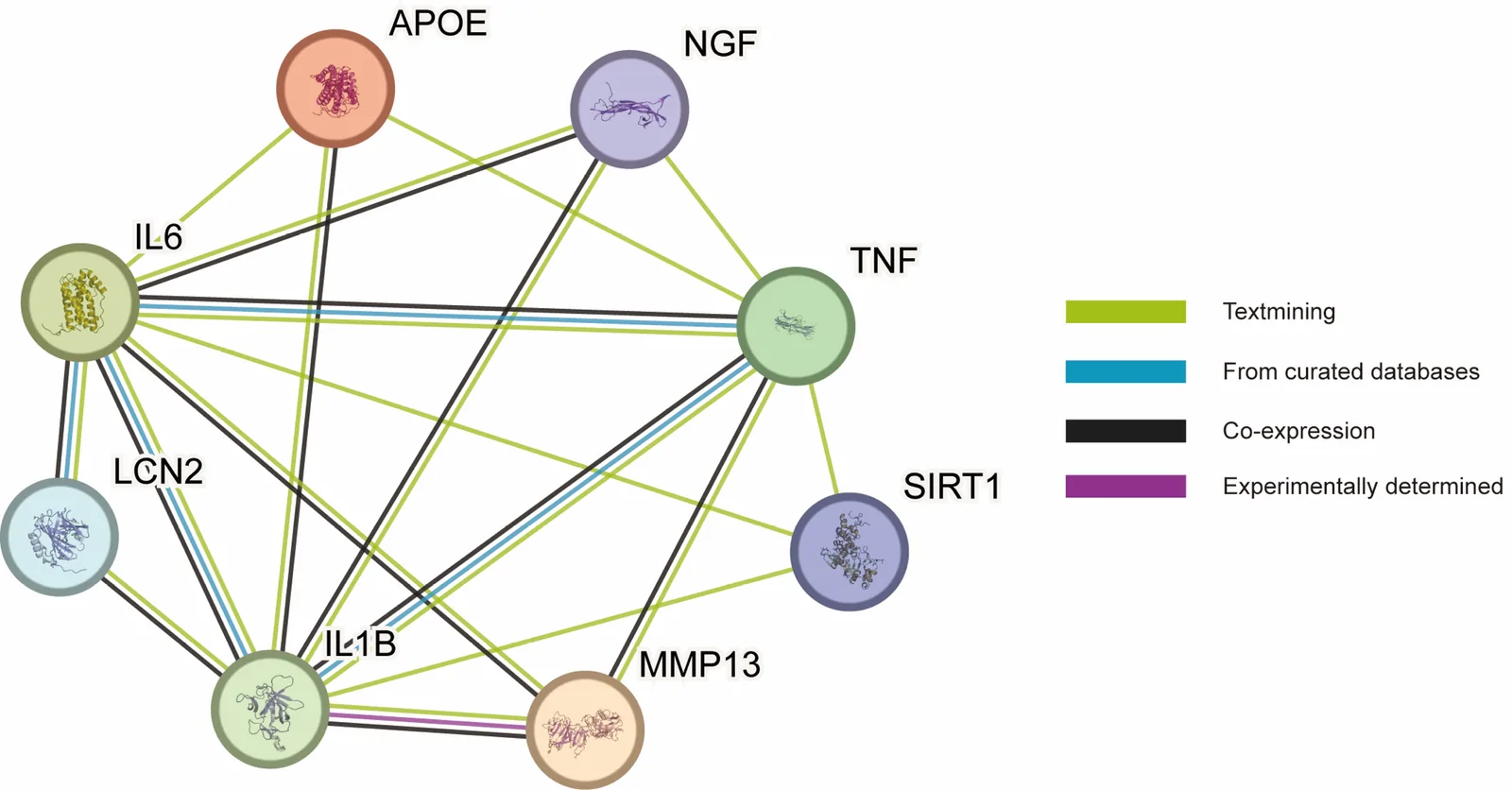
PPI network analysis of APOE and LBP-associated targets. The PPI network was constructed using the STRING database with a high-confidence threshold (interaction score ≥0.70). The central red node represents APOE (the identified causal protein). The surrounding blue nodes represent 7 established LBP/IDD-related biomarkers and drug targets: MMP13, IL-6, IL-1β, TNF-α, LCN2, SIRT1, and NGF. Edges (connecting lines) indicate high-confidence functional interactions supported by text mining, experimental evidence, or curated databases. The direct connections between APOE and key pro-inflammatory mediators (TNF, IL-6, and IL-1β) suggest that APOE may exert its protective effect against LBP through modulation of core inflammatory pathways.

To investigate potential functional relationships between APOE and known LBP-associated proteins, we constructed a PPI network. Results revealed high-confidence interactions (interaction score >0.70) between APOE and key inflammatory factors including TNF, IL-6, and IL-1β. These interactions were supported by multiple literature reports through text mining, suggesting APOE may participate in LBP pathogenesis by modulating these core inflammatory mediators (Fig. 5).

### 3.6. Druggability evaluation results

**Figure 6.**
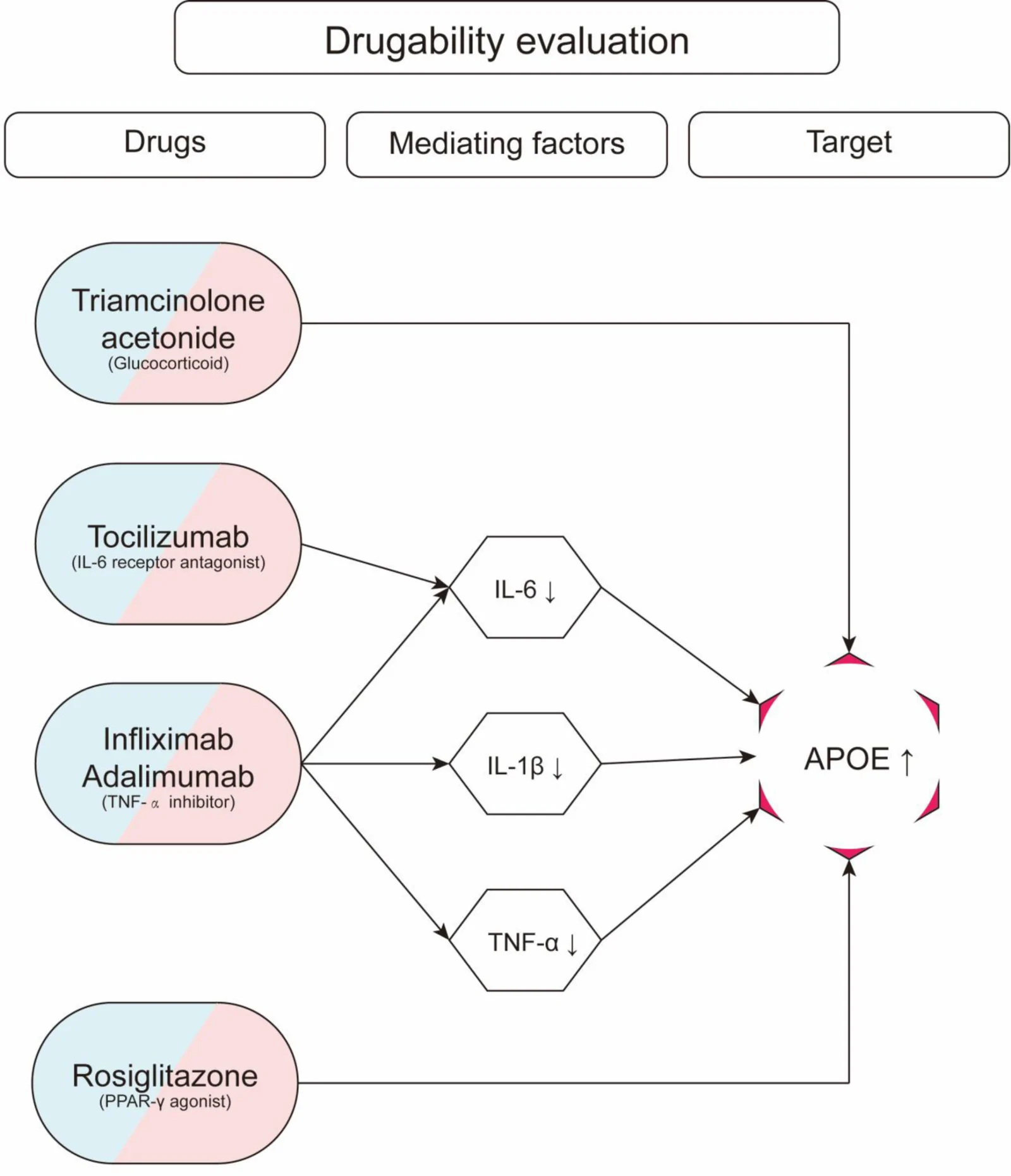
Results of Druggability Evaluation. The figure summarizes 3 classes of clinically approved or investigated pharmacological agents that can modulate APOE expression or activity, identified via DGIdb database screening and systematic literature mining. Glucocorticoids (e.g., Triamcinolone acetonide) directly upregulate endogenous APOE transcription via glucocorticoid receptor binding. PPARγ agonists (e.g., Rosiglitazone) indirectly enhance APOE synthesis through adipocyte-mediated pathways. Inflammatory cytokine receptor antagonists, including the IL-6 receptor blocker Tocilizumab and TNF-α inhibitors (Infliximab, Adalimumab), indirectly restore endogenous APOE levels by suppressing downstream inflammatory cascades. These findings provide repurposing leads for APOE-targeted early intervention in disc degeneration. APOE = Apolipoprotein E, LBP = low back pain, PPI = protein-protein interaction.

Comprehensive screening of the DGIdb database (v5.0.3) and literature evidence identified 3 therapeutic classes modulating APOE expression:Biologics: including the IL-6 receptor antagonist Tocilizumab and TNF-α inhibitors (Infliximab, Adalimumab): which indirectly restore APOE levels by suppressing inflammatory cascades (TNF-α/IL-6 signaling); PPARγ agonists (Rosiglitazone) that indirectly upregulate APOE synthesis via PPARγ pathway activation; and Glucocorticoids (Triamcinolone acetonide) which directly enhance APOE gene transcription. These findings provide pivotal drug repurposing leads for APOE-targeted therapies against LBP, particularly for countering chronic inflammation in IDD (Fig. 6).

## 4. Discussion

Low back pain, a prevalent spinal disorder, substantially compromises patients’ quality of life and imposes a significant socioeconomic burden worldwide. Despite advances in multimodal intervention strategies, current therapeutic options remain suboptimal for many patients due to the complex and heterogeneous nature of LBP. Consequently, identifying novel therapeutic targets for LBP holds significant clinical value.

We acknowledge that LBP is a clinically heterogeneous condition. In this study, we employed a combined phenotype from the FinnGen database that includes both nonspecific LBP (M54.5) and disc disorders with radiculopathy (M51.1). While this broad definition may capture diverse underlying pathologies, it aligns with our subsequent mechanistic focus on IDD, which constitutes a major pathological basis for the included diagnostic codes. The use of a combined phenotype in our primary analysis represents a trade-off between statistical power and phenotypic specificity, a limitation that we have considered in our interpretation.

To overcome the inherent confounding and reverse causation limitations of observational studies, we employed a two-sample MR framework integrating plasma proteomics cis-pQTL data with LBP GWAS summary statistics from the FinnGen database. Our analysis incorporated several rigorous quality control measures to ensure robust causal inference: exclusive use of European-ancestry samples to minimize population stratification bias; restriction to strong instruments with *F*-statistics >10 to mitigate weak instrument bias; and limitation to cis-pQTLs to reduce horizontal pleiotropy, thereby validating the exclusion restriction assumption. Robustness was further evaluated through Bayesian colocalization and Steiger directionality testing. Through this integrated approach, we identified APOE as a potential therapeutic target for LBP intervention.

LBP exhibits complex and multifactorial etiological mechanisms, with established evidence identifying IDD as its primary pathogenic factor.^[28]^IDD represents a multifactorial progressive degenerative disorder pathologically characterized by structural deterioration, including extracellular matrix (ECM) degradation, nucleus pulposus dehydration, and annulus fibrosus rupture, and functional dysregulation such as pro-inflammatory microenvironment formation. The disease core involves disrupted disc tissue homeostasis driven by multifactorial interplay of biomechanical stress, metabolic dysregulation, inflammatory cascades, and cellular senescence.^[29,30]^Recent evidence further reveals synergistic regulation through 4 molecular mechanisms: mitochondrial dysfunction from oxidative stress;^[31]^ lipid peroxidation damage via ferroptosis;^[32]^ inflammasome activation mediated by pyroptosis;^[33]^and lipotoxicity-induced metabolic disturbance.^[34]^These mechanisms converge through shared inflammatory pathways (NF-κB and NLRP3 signaling), amplifying into cytokine storms that release pro-inflammatory factors (IL-1β, TNF-α, etc), ultimately driving characteristic pathological changes: matrix degradation and disc cell apoptosis.^[35]^Critically, chronic inflammatory signaling constitutes the central pathological axis of IDD progression. Evidence indicates that during IDD progression, activated nucleus pulposus cells secrete abundant pro-inflammatory factors, including tumor necrosis factor-α (TNF-α) and interleukin family members (IL-1β, IL-6). These mediators activate key signaling pathways such as NF-κB, triggering aberrant overexpression of matrix metalloproteinases (MMPs) and a disintegrin and metalloproteinase with thrombospondin motifs (ADAMTS). By specifically degrading ECM components, these proteolytic enzymes progressively disrupt disc structural integrity and biomechanical function.^[33]^ Concurrently, inflammatory factors suppress ECM synthesis by inhibiting critical anabolic enzymes (SOX9), further depleting ECM and accelerating disc degeneration.^[36]^Moreover, these cytokines exacerbate IDD through dual mechanisms: inducing programmed cell death via activation of executioner caspases (caspase-3)^[37]^and triggering pyroptosis through NLRP3 inflammasome pathway activation.^[38]^Notably, senescent cells release substantial damage-associated molecular patterns during cell death, activating Toll-like receptor signaling pathways in neighboring cells. This establishes a vicious cycle of “inflammatory cytokine release → cell death → damage-associated molecular patterns rerelease” that amplifies inflammatory cascades: a key molecular driver of progressive IDD.^[39]^Consequently, interrupting these inflammatory pathways to suppress intracellular inflammation represents a promising therapeutic strategy for retarding IDD progression and treating LBP.

APOE serves as a critical lipid transporter in humans, with predominant expression in the liver and brain. It plays essential roles in regulating triglyceride and cholesterol metabolism while participating in certain inflammatory regulation processes.^[40,41]^Current research has extensively characterized APOE’s biological functions in the central nervous system, where it demonstrates significant clinical value, particularly in Alzheimer’s disease therapeutics.^[42]^This evidence confirms that APOE expression and functional activity can be effectively modulated pharmacologically, establishing it as a promising therapeutic target. However, compared to its extensively studied role in neurological disorders, the pathophysiological functions and therapeutic potential of APOE in spinal conditions, particularly IDD and LBP, have not been fully elucidated. Our MR analysis revealed a significant negative association between APOE and LBP risk (OR = 0.86, 95% CI: 0.81–0.91). This OR corresponds to a relative odds reduction of approximately 14% per 1 SD increase in genetically predicted plasma APOE levels (calculated as (1–OR) × 100%), a standard approach for interpreting protective odds ratios in epidemiological research.^[43]^ The corresponding 95% confidence interval (0.81–0.91) suggests a risk reduction ranging from approximately 9% to 19%, identifying APOE as an independent protective factor against LBP. This conclusion is corroborated by Zhou et al,^[44]^ who observed accelerated IDD in APOE-knockout mice. Complementary evidence from rabbit models further supports this finding.^[45]^Collectively, these animal studies substantiate APOE’s substantial protective role in LBP pathogenesis mediated by IDD. Further literature exploration suggests that APOE’s protective effects against LBP may operate through suppression of disc inflammation. A pivotal study revealed that macrophage-derived APOE drives anti-inflammatory polarization by modulating extracellular vesicle cargo composition, thereby promoting the CPT1A/fatty acid oxidation pathway while inhibiting NF-κB inflammatory signaling: ultimately exerting antioxidant and anti-inflammatory effects.^[46]^During this process, APOE upregulates miR-146a-5p expression while downregulating miR-142a-3p. The elevated miR-146a-5p suppresses NF-κB activation, reducing pro-inflammatory cytokine release (TNF, IL-6),^[47]^ whereas decreased miR-142a-3p activates the CPT1A/fatty acid oxidation pathway to enhance mitochondrial function, boost intracellular fatty acid oxidation, and mitigate oxidative stress.^[48]^Macrophages constitute pivotal cellular mediators in IDD, closely associated with inflammasome activation during degenerative progression.^[49]^APOE likely confers protection against LBP by orchestrating anti-inflammatory macrophage polarization: thereby suppressing inflammatory responses and slowing disc degeneration.

Building upon these findings, we substantiate APOE’s protective role against LBP, likely mediated through suppression of inflammatory pathways and mitigation of oxidative stress: thereby attenuating systemic inflammatory responses. Consequently, elevating endogenous APOE levels in LBP patients to block or suppress inflammation during disc degeneration represents a promising therapeutic strategy for IDD-related LBP. Through systematic DGIdb screening, we identified triamcinolone acetonide as a potent upregulator of endogenous APOE. As a glucocorticoid agent, triamcinolone acetonide binds intracellular glucocorticoid receptors (GRs) to exert broad-spectrum anti-inflammatory effects, currently primarily used for treating inflammatory macular edema.^[50]^Animal studies by Hitoshi Chiba et al demonstrated significantly increased plasma APOE levels in mice following triamcinolone acetonide administration,^[51]^ suggesting its therapeutic potential for LBP, though further experimental validation remains necessary. Beyond glucocorticoids, PPARγ agonists also show promise for modulating endogenous APOE in LBP treatment. Current evidence indicates these agonists upregulate APOE expression through adipocyte-mediated pathways, although the precise mechanisms require elucidation.^[52]^Additionally, IL-6 receptor antagonists (Tocilizumab) and TNF-α inhibitors (Infliximab) may indirectly sustain endogenous APOE levels by suppressing inflammatory cytokine secretion (TNF, ILs) and blocking downstream signaling cascades.^[53,54]^

In summary, a significant causal relationship exists between APOE and LBP. APOE functions as a protective factor against LBP and holds promise as a therapeutic target for disc degeneration-mediated LBP. Endogenous APOE expression can be upregulated through glucocorticoids, PPARγ agonists, or inflammatory cytokine receptor antagonists. Nevertheless, our study has limitations: the exclusive use of European-ancestry samples may limit generalizability to other populations, and the precise mechanisms by which certain drugs exert therapeutic effects via APOE modulation require further investigation.

## Acknowledgements

This research was supported by The Project of Zhejiang provincial plan for TCM science and technology (2025ZR053); Key Discipline of Traditional Chinese Medicine in Zhejiang Province (2024-XK-57); The Construction Fund of Key Medical Discipline of Hangzhou (2025HZZD16). The funders had no role in study design, data collection and analysis, decision to publish, or preparation of the manuscript.

## Author contributions

**Conceptualization:** Jianye Yang, Weihui Qi.

**Methodology:** Jianye Yang, Weihui Qi, Zhenglin Mei.

**Visualization:** Zhenglin Mei.

**Writing – original draft:** Jianye Yang, Weihui Qi.

**Writing – review & editing:** Dong Wang, Hao Pan, Yongjiang He.





## References

[1] Knezevic NN Candido KD Vlaeyen JWS Van Zundert J Cohen SP. Low back pain. Lancet. 2021;398:78–92. 34115979 10.1016/S0140-6736(21)00733-9

[2] Hartvigsen J Hancock MJ Kongsted A; Lancet Low Back Pain Series Working Group. What low back pain is and why we need to pay attention. Lancet. 2018;391:2356–67. 29573870 10.1016/S0140-6736(18)30480-X

[3] Wu A March L Zheng X. Global low back pain prevalence and years lived with disability from 1990 to 2017: estimates from the Global Burden of Disease Study 2017. Ann Transl Med. 2020;8:299. 32355743 10.21037/atm.2020.02.175 PMC7186678

[4] Dieleman JL Cao J Chapin A. US health care spending by payer and health condition, 1996-2016. JAMA. 2020;323:863–84. 32125402 10.1001/jama.2020.0734 PMC7054840

[5] George SZ Fritz JM Silfies SP. Interventions for the management of acute and chronic low back pain: revision 2021. J Orthop Sports Phys Ther. 2021;51:CPG1–CPG60. 10.2519/jospt.2021.0304 PMC10508241 34719942

[6] Chou R. Low back pain. Ann Intern Med. 2021;174:ITC113–28. 34370518 10.7326/AITC202108170

[7] Webster LR Markman J. Medical management of chronic low back pain: efficacy and outcomes. Neuromodulation. 2014;17(Suppl 2):18–23. 25395113 10.1111/j.1525-1403.2012.00496.x

[8] Mu J Furlan AD Lam WY Hsu MY Ning Z Lao L. Acupuncture for chronic nonspecific low back pain. Cochrane Database Syst Rev. 2020;12:CD013814. 33306198 10.1002/14651858.CD013814 PMC8095030

[9] Suhre K Arnold M Bhagwat AM. Connecting genetic risk to disease end points through the human blood plasma proteome. Nat Commun. 2017;8:14357. 28240269 10.1038/ncomms14357 PMC5333359

[10] Khan AN Jacobsen HE Khan J. Inflammatory biomarkers of low back pain and disc degeneration: a review. Ann N Y Acad Sci. 2017;1410:68–84. 29265416 10.1111/nyas.13551 PMC5744892

[11] Mao R Li J Xiao W. Identification of prospective aging drug targets via Mendelian randomization analysis. Aging Cell. 2024;23:e14171. 38572516 10.1111/acel.14171 PMC11258487

[12] Bowden J Holmes MV. Meta-analysis and Mendelian randomization: a review. Res Synth Methods. 2019;10:486–96. 30861319 10.1002/jrsm.1346 PMC6973275

[13] Tachmazidou I Hatzikotoulas K Southam L; arcOGEN Consortium. Identification of new therapeutic targets for osteoarthritis through genome-wide analyses of UK Biobank data. Nat Genet. 2019;51:230–6. 30664745 10.1038/s41588-018-0327-1 PMC6400267

[14] Zhang J Dutta D Köttgen A; CKDGen Consortium. Plasma proteome analyses in individuals of European and African ancestry identify cis-pQTLs and models for proteome-wide association studies. Nat Genet. 2022;54:593–602. 35501419 10.1038/s41588-022-01051-w PMC9236177

[15] Kurki MI Karjalainen J Palta P; FinnGen. FinnGen provides genetic insights from a well-phenotyped isolated population. Nature. 2023;613:508–18. 36653562 10.1038/s41586-022-05473-8 PMC9849126

[16] Burgess S Butterworth A Thompson SG. Mendelian randomization analysis with multiple genetic variants using summarized data. Genet Epidemiol. 2013;37:658–65. 24114802 10.1002/gepi.21758 PMC4377079

[17] Giambartolomei C Vukcevic D Schadt EE. Bayesian test for colocalisation between Pairs of genetic association studies using summary statistics. PLoS Genet. 2014;10:e1004383. 24830394 10.1371/journal.pgen.1004383 PMC4022491

[18] Burgess S Davey Smith G Davies NM. Guidelines for performing Mendelian randomization investigations: update for summer 2023. Wellcome Open Res. 2019;4:186. 32760811 10.12688/wellcomeopenres.15555.1 PMC7384151

[19] Hemani G Tilling K Davey Smith G. Orienting the causal relationship between imprecisely measured traits using GWAS summary data. PLoS Genet. 2017;13:e1007081. 29149188 10.1371/journal.pgen.1007081 PMC5711033

[20] Inada M Wang Y Byrne MH. Critical roles for collagenase-3 (Mmp13) in development of growth plate cartilage and in endochondral ossification. Proc Natl Acad Sci USA. 2004;101:17192–7. 15563592 10.1073/pnas.0407788101 PMC535367

[21] Lim YZ Wang Y Cicuttini FM. Association between inflammatory biomarkers and nonspecific low back pain: a systematic review. Clin J Pain. 2020;36:379–89. 31990692 10.1097/AJP.0000000000000810

[22] Wang Y Che M Xin J Zheng Z Li J Zhang S. The role of IL-1β and TNF-α in intervertebral disc degeneration. Biomed Pharmacother. 2020;131:110660. 32853910 10.1016/j.biopha.2020.110660

[23] Fan C Wang W Yu Z. M1 macrophage-derived exosomes promote intervertebral disc degeneration by enhancing nucleus pulposus cell senescence through LCN2/NF-κB signaling axis. J Nanobiotechnology. 2024;22:301. 38816771 10.1186/s12951-024-02556-8 PMC11140985

[24] Wei Y-F Zhang H-L Li L-Z. Sirt1 blocks nucleus pulposus and macrophages crosstalk by inhibiting RelA/Lipocalin 2 axis. J Orthop Translat. 2025;50:30–43. 39758288 10.1016/j.jot.2024.11.008 PMC11699611

[25] Rizzo RRN Ferraro MC Wewege MA. Targeting neurotrophic factors for low back pain and sciatica: a systematic review and meta-analysis. Rheumatology (Oxford). 2022;61:2243–54. 34677587 10.1093/rheumatology/keab785

[26] You Y Li J Zhang Y. Exploring the role of β2-microglobulin in the relationship between physical activity and DNAm-predicted PhenoAge: evidence from a population-based and mice single-cell RNA-sequencing study. J Adv Res. 2026;86:743–55. 41285310 10.1016/j.jare.2025.11.047 PMC13453862

[27] You Y Li J Zhang Y Li X Li X Ma X. Exploring the potential relationship between short sleep risks and cognitive function from the perspective of inflammatory biomarkers and cellular pathways: insights from population-based and mice studies. CNS Neurosci Ther. 2024;30:e14783. 38797980 10.1111/cns.14783 PMC11128714

[28] Yang S Zhang F Ma J Ding W. Intervertebral disc ageing and degeneration: the antiapoptotic effect of estrogen. Ageing Res Rev. 2020;57:100978. 31669486 10.1016/j.arr.2019.100978

[29] Guo W Li B-L Zhao J-Y Li X-M Wang L-F. Causal associations between modifiable risk factors and intervertebral disc degeneration. Spine J. 2024;24:195–209. 37939919 10.1016/j.spinee.2023.10.021

[30] Wang F Cai F Shi R Wang XH Wu XT. Aging and age related stresses: a senescence mechanism of intervertebral disc degeneration. Osteoarthritis Cartilage. 2016;24:398–408. 26455958 10.1016/j.joca.2015.09.019

[31] Wang Y Cheng H Wang T Zhang K Zhang Y Kang X. Oxidative stress in intervertebral disc degeneration: molecular mechanisms, pathogenesis and treatment. Cell Prolif. 2023;56:e13448. 36915968 10.1111/cpr.13448 PMC10472537

[32] Yang RZ Xu WN Zheng HL. Involvement of oxidative stress‐induced annulus fibrosus cell and nucleus pulposus cell ferroptosis in intervertebral disc degeneration pathogenesis. J Cell Physiol. 2020;236:2725–39. 32892384 10.1002/jcp.30039 PMC7891651

[33] Zhou K-S Ran R Gong C-Y. Roles of pyroptosis in intervertebral disc degeneration. Pathol Res Pract. 2023;248:154685. 37494803 10.1016/j.prp.2023.154685

[34] Tu S Dong Y Li C. Phosphatidylcholine ameliorates palmitic acid-induced lipotoxicity by facilitating endoplasmic reticulum and mitochondria contacts in intervertebral disc degeneration. JOR Spine. 2025;8:e70062. 40171442 10.1002/jsp2.70062 PMC11956213

[35] Shamji MF Setton LA Jarvis W. Proinflammatory cytokine expression profile in degenerated and herniated human intervertebral disc tissues. Arthritis Rheum. 2010;62:1974–82. 20222111 10.1002/art.27444 PMC2917579

[36] Gruber HE Norton HJ Ingram JA Hanley EN. The SOX9 transcription factor in the human disc: decreased immunolocalization with age and disc degeneration. Spine (Phila Pa 1976). 2005;30:625–30. 15770176 10.1097/01.brs.0000155420.01444.c6

[37] Jiang M Qi L Li L Li Y. The caspase-3/GSDME signal pathway as a switch between apoptosis and pyroptosis in cancer. Cell Death Discov. 2020;6:112. 33133646 10.1038/s41420-020-00349-0 PMC7595122

[38] He Y Hara H Núñez G. Mechanism and regulation of NLRP3 inflammasome activation. Trends Biochem Sci. 2016;41:1012–21. 27669650 10.1016/j.tibs.2016.09.002 PMC5123939

[39] Vasudevan SO Behl B Rathinam VA. Pyroptosis-induced inflammation and tissue damage. Semin Immunol. 2023;69:101781. 37352727 10.1016/j.smim.2023.101781 PMC10598759

[40] Almeida FC Patra K Giannisis A. APOE genotype dictates lipidomic signatures in primary human hepatocytes. J Lipid Res. 2024;65:100498. 38216055 10.1016/j.jlr.2024.100498 PMC10875595

[41] Sun Y Zhu D Kong L. Vasicine attenuates atherosclerosis via lipid regulation, inflammation inhibition, and autophagy activation in ApoE-/- mice. Int Immunopharmacol. 2024;142:112996. 39243558 10.1016/j.intimp.2024.112996

[42] Serrano-Pozo A Das S Hyman BT. APOE and Alzheimer’s disease: advances in genetics, pathophysiology, and therapeutic approaches. Lancet Neurol. 2021;20:68–80. 33340485 10.1016/S1474-4422(20)30412-9 PMC8096522

[43] Bala MM Agarwal A Klatt KC. Nutrition users’ guides: RCTs part 2 - structured guide for interpreting and applying study results from randomised controlled trials on therapy or prevention questions. BMJ Nutr Prev Health. 2024;7:e000834. 10.1136/bmjnph-2023-000834 PMC11773662 39882290

[44] Zhou Y Chen X Tian Q. Deletion of ApoE leads to intervertebral disc degeneration via aberrant activation of adipokines. Spine (Phila Pa 1976). 2022;47:899–907. 34919078 10.1097/BRS.0000000000004311

[45] Beierfuß A Hunjadi M Ritsch A Kremser C Thomé C Mern DS. APOE-knockout in rabbits causes loss of cells in nucleus pulposus and enhances the levels of inflammatory catabolic cytokines damaging the intervertebral disc matrix. PLoS One. 2019;14:e0225527. 31751427 10.1371/journal.pone.0225527 PMC6871866

[46] Phu TA Ng M Vu NK Gao AS Raffai RL. ApoE expression in macrophages communicates immunometabolic signaling that controls hyperlipidemia-driven hematopoiesis & inflammation via extracellular vesicles. J Extracell Vesicles. 2023;12:e12345. 37593979 10.1002/jev2.12345 PMC10436255

[47] Olivieri F Prattichizzo F Giuliani A. miR-21 and miR-146a: the microRNAs of inflammaging and age-related diseases. Ageing Res Rev. 2021;70:101374. 34082077 10.1016/j.arr.2021.101374

[48] Phu TA Vu NK Ng M. ApoE enhances mitochondrial metabolism via microRNA-142a/146a-regulated circuits that suppress hematopoiesis and inflammation in hyperlipidemia. Cell Rep. 2023;42:113206. 37824329 10.1016/j.celrep.2023.113206

[49] Latz E Xiao TS Stutz A. Activation and regulation of the inflammasomes. Nat Rev Immunol. 2013;13:397–411. 23702978 10.1038/nri3452 PMC3807999

[50] Fung S Syed YY. Suprachoroidal space triamcinolone acetonide: a review in uveitic macular edema. Drugs. 2022;82:1403–10. 36018461 10.1007/s40265-022-01763-7 PMC9512860

[51] Chiba H Akita H Hui SP. Effects of triamcinolone on brain and cerebrospinal fluid apolipoprotein E levels in rats. Life Sci. 1997;60:1757–61. 9150415 10.1016/s0024-3205(97)00135-5

[52] Huang ZH Reardon CA Mazzone T. Endogenous ApoE expression modulates adipocyte triglyceride content and turnover. Diabetes. 2006;55:3394–402. 17130485 10.2337/db06-0354

[53] Kang S Kishimoto T. Interplay between interleukin-6 signaling and the vascular endothelium in cytokine storms. Exp Mol Med. 2021;53:1116–23. 34253862 10.1038/s12276-021-00649-0 PMC8273570

[54] Murdaca G Spanò F Contatore M. Immunogenicity of infliximab and adalimumab: what is its role in hypersensitivity and modulation of therapeutic efficacy and safety? Expert Opin Drug Saf. 2016;15:43–52. 26559805 10.1517/14740338.2016.1112375

